# Control of box C/D snoRNP assembly by N^6^‐methylation of adenine

**DOI:** 10.15252/embr.201743967

**Published:** 2017-06-16

**Authors:** Lin Huang, Saira Ashraf, Jia Wang, David MJ Lilley

**Affiliations:** ^1^ Cancer Research UK Nucleic Acid Structure Research Group The University of Dundee Dundee UK

**Keywords:** epigenetics, G·A base pairs, k‐turn, RNA methylation, signal recognition particle, RNA Biology, Structural Biology

## Abstract

N^6^‐methyladenine is the most widespread mRNA modification. A subset of human box C/D snoRNA species have target GAC sequences that lead to formation of N^6^‐methyladenine at a key *trans* Hoogsteen‐sugar A·G base pair, of which half are methylated *in vivo*. The GAC target is conserved only in those that are methylated. Methylation prevents binding of the 15.5‐kDa protein and the induced folding of the RNA. Thus, the assembly of the box C/D snoRNP could in principle be regulated by RNA methylation at its critical first stage. Crystallography reveals that N^6^‐methylation of adenine prevents the formation of *trans* Hoogsteen‐sugar A·G base pairs, explaining why the box C/D RNA cannot adopt its kinked conformation. More generally, our data indicate that sheared A·G base pairs (but not Watson–Crick base pairs) are more susceptible to disruption by N^6^mA methylation and are therefore possible regulatory sites. The human signal recognition particle RNA and many related Alu retrotransposon RNA species are also methylated at N6 of an adenine that forms a sheared base pair with guanine and mediates a key tertiary interaction.

## Introduction

All cellular RNA is subject to dynamic covalent modification, and posttranscriptional modification of RNA is diverse and widespread 1, 2. N^6^‐methyladenine is the most common modification in RNA 3, 4, 5. It is found in mRNA (a typical eukaryotic mRNA will have several such methylated adenine nucleotides), as well as in lncRNA species such as Xist 4, 5 and MALAT1 6, 7) and in viral RNA 8. This epigenetic marker has proposed roles in the modulation of RNA stability 9, control of translation efficiency 10, 11, and in gene regulation 12, and N^6^‐methyladenine is most frequently observed in regions of mRNA indicative of control functions 5. The level of modification is subject to regulation by methyl transferase 13 and demethylase 14, 15 enzymes, and they have been linked to human disease 15, 16, 17, 18.

**Figure 1:**
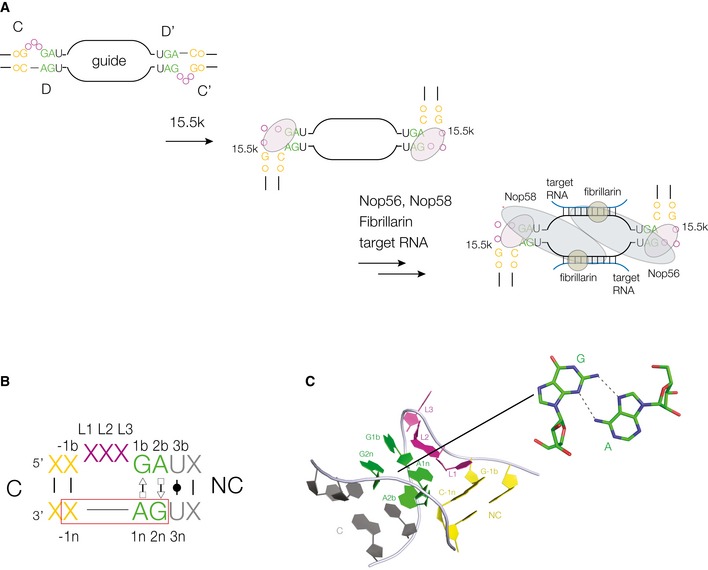
Assembly of box C/D snoRNA, and their k‐turn structures Scheme depicting the assembly of box C/D snoRNP. The first stage is the binding of the 15.5k protein to the box C/D and C’/D’ k‐turns.The general sequence of a k‐turn, with the standard nomenclature of nucleotide positions. Note that when the ‐1n nucleotide is cytosine, this generates a GAC sequence on the non‐bulged strand (boxed) that is a potential target for N^6^‐methylation of the conserved adenine at the 1n position. The two helical arms of the k‐turn are named C (canonical) and NC (non‐canonical) as indicated.Structure of a standard box C/D k‐turn, together with the chemical structure of the G1b·A1n and A2b·G2n *trans* sugar‐Hoogsteen G·A base pairs (PDB 1RLG 74).

In this work, we have uncovered a putative role of epigenetic regulation in a subset of human snoRNP complexes that are involved in the site‐specific modification of RNA. In archaea and the eukaryotes, the box C/D snoRNP complexes direct the site‐specific 2′‐*O*‐methylation of rRNA and tRNA by providing complementary guide RNA for specificity and a SAM‐dependent methyl transferase enzyme to modify the target ribose 19, 20, 21, 22, 23, 24, 25, 26. The complex is equivalent to a bulged RNA duplex, where both 12 nucleotide strands of the central region are the guide sequences that hybridize to the target RNA molecules to be methylated (Fig 1A). The conserved box C/D and C’/D’ sequences are located in the duplexes that flank the guide region, and both adopt the k‐turn conformation (k‐turn structure is reviewed in 27). A series of proteins assembles on the snoRNA 28, 29, 30, 31, 32. In the first step, a member of the L7Ae family, the 15.5‐kDa protein (15.5k) in humans, binds to each of the k‐turns. This is followed by a Nop58 binding to box C/D and Nop56 binding to box C’/D’, associating through a coiled‐coil domain to form a heterodimer. Finally, two molecules of the methyl transferase fibrillarin associate with the complex to generate the catalytically active snoRNP. Thus, the key event that initiates the assembly of the complex is the association between the 15.5‐kDa protein and the k‐turn. In particular, Watkins *et al* 30 showed that if the binding of 15.5k to the human box C/D k‐turn is prevented by sequence changes that are known to disrupt k‐turn folding, this blocks assembly of the box C/D snoRNP.

Binding of L7Ae‐family proteins induces the formation of the tightly kinked k‐turn structure 33, 34, 35 (Fig 1B). The core of the k‐turn structures formed by the box C/D and C'/D’ elements comprises consecutive *trans* sugar‐Hoogsteen G·A base pairs (sometimes termed sheared base pairs) that position the conserved adenine nucleobases to make key cross‐strand hydrogen bonds that stabilize the conformation (Fig 1C). In general, k‐turns fall into two classes depending on whether or not they will fold in response to the presence of metal ions 36. We have noted that box C/D k‐turns remain unfolded under these conditions, and so require protein‐induced folding. Once this has been achieved the assembly of the snoRNP can proceed to form the active methylation complex. However, a process that blocks the folding of the k‐turn would likely prevent the assembly of a functional snoRNP complex.

While the majority of k‐turn structures have a C‐G base pair at the ‐1b,‐1n position, we have noted that in the box C/D k‐turn sequences this base pair is sometimes inverted, so that the ‐1n nucleotide is a cytosine. Since the highly conserved core of the k‐turn requires 2n = G and 1n = A, this creates a GAC sequence on the non‐bulged strand (boxed in Fig 1B) that is a putative target for methylation of adenine N6 9. Given the critical role of the A1n nucleobase in the folded k‐turn, it seemed highly probable that N^6^‐methylation would interfere with folding. We have therefore studied the occurrence of ‐1n = C in human box C/D and C’/D’ sequences. We find that a significant subset have ‐1n = C, about half of which are known to be methylated, and that those that are methylated are strongly conserved. We further show here that N^6^‐methyladenine at the 1n position of box C/D and C’/D’ k‐turns prevents specific binding of the 15.5k protein, and consequent induced folding, and use X‐ray crystallography to provide a structural explanation for this. Indeed, our structural analysis indicates that sheared A·G base pairs are much more sensitive to N^6^‐methylation of adenine compared to Watson–Crick base pairs, and we show that these effects extend to other structures involving such A·G base pairs.

## Results

### N^6^‐adenine methylation of box C/D sequences

**Figure 2:**
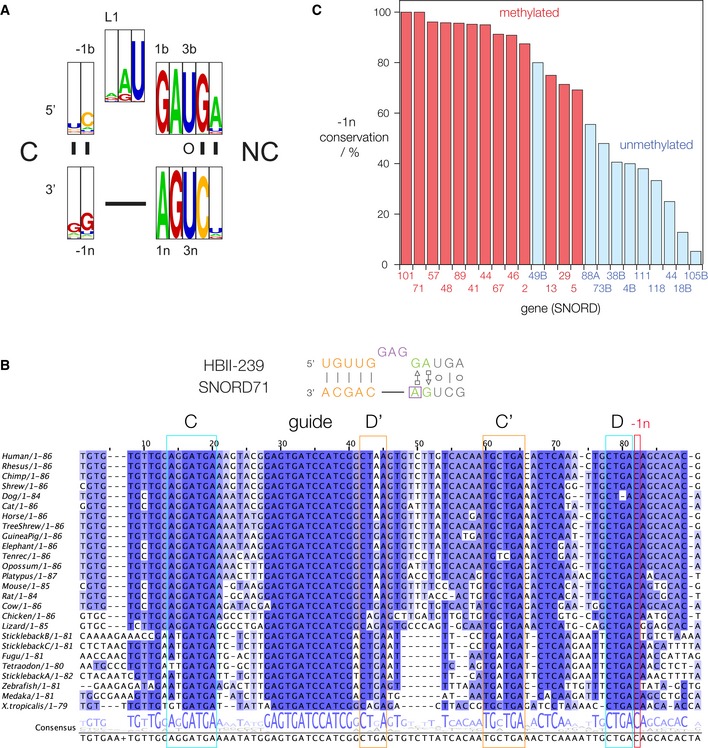
Bioinformatic analysis of box C/D and C’/D’ methylation in the human genome WebLogo plot showing the occurrence of box C/D sequences in human snoRNA. The frequency of distribution for the ‐1b,‐1n pair is CG 65.27%; AU 8.78%; UA 4.96%; UG 4.20%; GC 3.82%.Sequence alignment for SNORD71. The boxes C, D, C’, and D’ are boxed, as is the ‐1n sequence.The conservation of ‐1n sequences (fraction of cytosine as percent) for eukaryotic box C/D and C’/D’ sequences for which ‐1n = C in humans. Those that are methylated in humans are colored red while those that are not are colored blue.

Analysis of the targets for methylation by the METTL3–METTL14 methyl transferase complex in eukaryotes shows the preferred sequence to be DRACH (where D denotes A, G, or U; R is A or G; and H is A, C, or U), with GAC as the most common site of methylation 4, 5. The box C/D and C’/D’ sequences adopt the k‐turn conformation on binding the 15.5‐kDa protein (15.5k), with the unbulged strand having GA at the 2n,1n sequence. In most other k‐turns, the ‐1n nucleotide is G, but in some box C/D‐type k‐turns it is C, thus creating a GAC sequence with the central adenine as a potential methylation target (Fig 2A). N^6^‐methylation of the A1n might be anticipated to affect the folding of the k‐turn into the kinked conformation. We therefore analyzed the occurrence of ‐1n = C in human box C/D and C’/D’ sequences (although many D’ boxes could not be reliably annotated either because of the short length of these snoRNA‐like genes, or the lack of evolutionary conservation or sequence motif signals).

**Figure 3:**
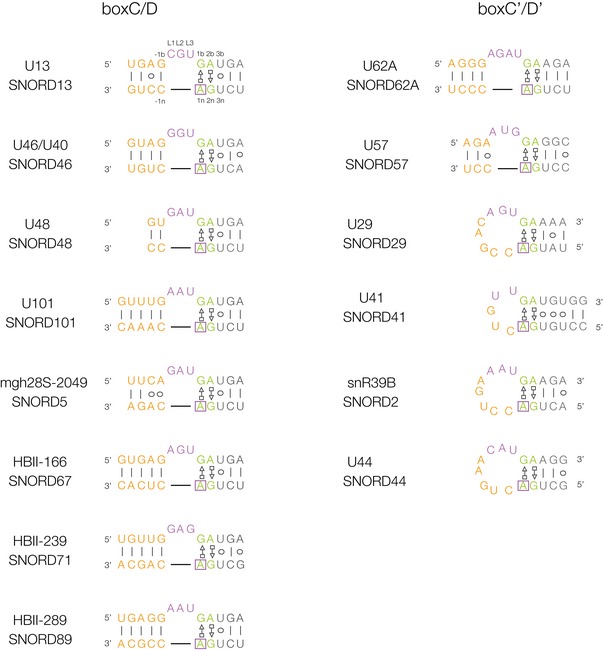
Human box C/D and C’/D’ demonstrated to be methylated *in vivo* In each case, the site of methylation is the A1n shown boxed. Note that the majority of the box C’/D’ sequences are k‐loop structures.

Using the snoRNABase 37 and snOPY 38 databases, we identified 27 human snoRNA sequences with 2n = G, 1n = A, and ‐1n = C, comprising 17 box D and 10 box D’. These are potential methylation targets, so we then examined the RMBase database 39, which contains single‐nucleotide or high‐resolution data on N^6^‐methyladenine sites 40, 41, to see whether any had been experimentally demonstrated to be methylated *in vivo*. From this, we identified 14 human sequences that were shown to be N^6^‐methylated at A1n (eight from box D and six from box D’) (Table 1, Fig 3). Most of these have been identified in more than one independent experiment. The A1n of SNORD13 from chromosome 8 was found to be methylated in 11 different sequencing experiments. Paralogs of SNORD13 and SNORD46 encoded on different chromosomal locations were found to be methylated.

**Table 1:** BoxC/D RNA A1n N^6^‐methylation sites identified from RMBase. In human, 19 unique positions been modified, comprising 14 unique boxC/D RNA (8 D box and 6 D’ box). In mouse, one D’ box was identified; importantly, this site is also modified in humans

Human	GeneName	modID	Chromosome	Position	Strand	SupportNum	Position in k‐turn
1	SNORD13	m6A_site_124853	chr8	33371093	+	11	boxD 1n
	SNORD13	m6A_site_136193	chrX	23525329	−	8	boxD 1n
	SNORD13	m6A_site_24525	chr11	66988484	+	3	boxD 1n
	SNORD13	m6A_site_130577	chr9	75142252	+	1	boxD 1n
2	SNORD46	m6A_site_4620	chr1	45242258	+	4	boxD 1n
	SNORD46	m6A_site_122222	chr7	132437879	+	2	boxD 1n
3	SNORD48	m6A_site_111371	chr6	31803100	+	4	boxD 1n
4	SNORD101	m6A_site_115173	chr6	133136512	+	1	boxD 1n
5	SNORD5	m6A_site_25652	chr11	93466398	−	2	boxD 1n
6	SNORD67	m6A_site_22376	chr11	46783944	−	3	boxD 1n
7	SNORD71	m6A_site_50014	chr16	71792313	−	2	boxD 1n
8	SNORD89	m6A_site_74416	chr2	101889404	−	1	boxD 1n
9	SNORD2	m6A_site_95922	chr3	186502617	+	1	boxD’ 1n
10	SNORD29	m6A_site_23425	chr11	62621408	−	1	boxD’ 1n
11	SNORD41	m6A_site_64597	chr19	12817310	−	1	boxD’ 1n
12	SNORD44	m6A_site_10668	chr1	173835140	−	9	boxD’ 1n
13	SNORD57	m6A_site_79190	chr20	2637610	+	1	boxD’ 1n
14	SNORD62A	m6A_site_133909	chr9	134361128	+	2	boxD’ 1n
	SNORD62B	m6A_site_133912	chr9	134365904	+	5	boxD’ 1n
Mouse	SNORD2	m6A_site_29280	chr16	23108986	+	1	boxD’ 1n

If the N^6^‐methylation of A1n in these k‐turn‐forming snoRNA species is functionally significant then we might expect that the ‐1n = C sequence that is required to generate the methylation target should be conserved in other eukaryotes. We therefore examined the conservation of the ‐1n position in other organisms using the snoRNABase and snOPY databases (Appendix Table S1). We found that the ‐1n sequence is highly conserved as C for those 14 sequences that are methylated *in vivo*. This is 100% conserved in SNORD71 and SNORD101. 26 SNORD71 sequences are conserved in vertebrates including human, rat, chicken, frog, and fugu (pufferfish). Outside the conserved C, D, C’, D’ and guide region, only the ‐1n C and its complementary paired base (‐1b = G) are 100% conserved (Fig 2B). In marked contrast, for 12 of 13 box C/D RNAs that are not methylated ‐1n is significantly less conserved as C. This is shown graphically in Fig 2C. Evidently, the ‐1n position of the snoRNA k‐turn is critical for function in the methylated sequences, but much less so in those that are not.

### N^6^‐methylation of adenine 1n interferes with 15.5k‐induced folding of box C/D and C’/D’ k‐turns

We have seen that in a subset of human box C/D k‐turn sequences the ‐1n position is a cytosine, creating a GAC methylation target on the non‐bulged strand. The METTL3‐METTL14 methyl transferase complex could therefore convert the A1n of the k‐turn to N^6^‐methyladenine. We know that k‐turn folding is most sensitive to modification of the G1b·A1n base pair, and we therefore set out to test whether or not N^6^‐methyladenine would impair folding of box C/D‐type k‐turns. In general, these k‐turn have sequences that are not susceptible to ion‐induced folding 36 so that they require the binding of protein to undergo folding into the kinked conformation. For the human snoRNA species this is the 15.5k protein. We examined the folding of representative human box C/D and C’/D’ k‐turns in response to addition of human 15.5k using two approaches. One was by electrophoretic migration in non‐denaturing polyacrylamide gels, where protein binding results in retardation of the RNA. The other is a spectroscopic method based upon fluorescence resonance energy transfer (FRET) between fluorescein and Cy3 fluorophores attached to the termini of a short snoRNA duplex with a central k‐turn motif; protein‐induced folding into the kinked conformation brings the fluorophores closer together, resulting in an increased efficiency of energy transfer (*E*_FRET_).

**Figure 4:**
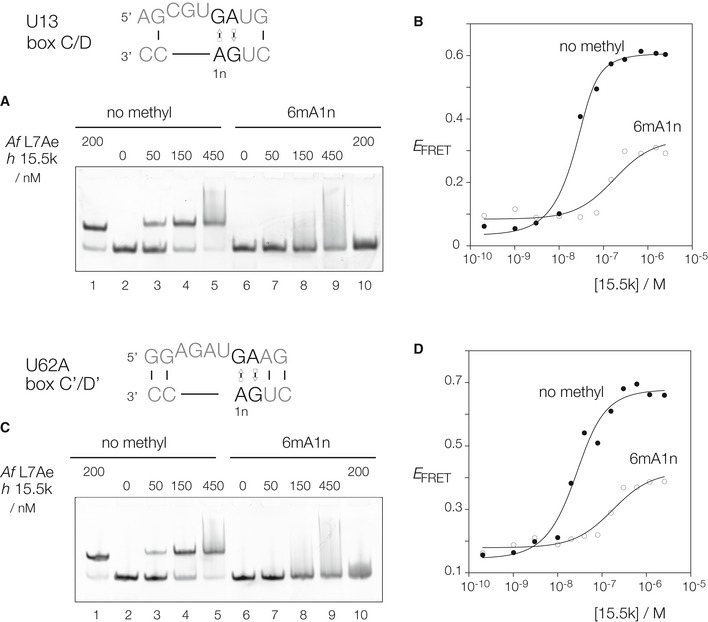
15.5‐kDa protein binding and induced folding of k‐turn conformation is blocked by N^6^‐methylation of adenine in box C/D and C’/D’ snoRNA A–DTwo human snoRNA k‐turns with ‐1n = C (thus creating a GAC methylation target on the non‐bulged strand) have been chosen as examples. Human 15.5k protein binding was studied by gel electrophoretic retardation analysis (A and C) and induced folding analyzed by FRET between fluorescein and Cy3 terminally attached to a short duplex (B and D). The chosen snoRNA species were the SNORD13 (U13) box C/D (A, B) and SNORD62A (U62A) box C’/D’ (C, D) k‐turns. Each species was studied with and without N^6^‐methylation at the A1n position. 200 nM RNA was incubated with the indicated concentration of 15.5k, or *A. fulgidus* L7Ae proteins and applied to 10% polyacrylamide gels electrophoresed under non‐denaturing conditions (A and C). Binding of either protein to the unmodified RNA (tracks 1 through 5) led to the formation of discrete retarded species. At higher concentrations of 15.5k, a continuous smear of complexes ran up the gel suggesting non‐specific binding beyond stoichiometric conditions. By contrast, no specific RNA–protein complexes were observed when N^6^‐methyladenine‐containing RNA was used (tracks 6 through 10). FRET efficiency (*E*_FRET_) was measured as a function of 15.5k concentration for non‐methylated RNA (closed circles) and N^6^‐methyladenine‐containing RNA (open circles). The data have been fitted to a simple binding model (line).

Results are shown for one box C/D k‐turn from SNORD13 (U13) snoRNA, and one box C’/D’ k‐turn from SNORD62A (U62A) snoRNA (Fig 4). The unmethylated RNA species both migrate as a discrete band of retarded mobility when incubated with either human 15.5k or *Archeoglobus fulgidus* L7Ae (*Af* L7Ae) protein (Fig 4A and C). At higher 15.5k concentrations, further retarded species are observed as a smear running up the gel, indicative of additional non‐specific binding. Over the same range of 15.5k protein concentration used in the electrophoresis, the fluorescence spectroscopy reveals an increase in energy transfer (final *E*_FRET_ = 0.6) indicative of a folding of the k‐turns (Fig 4B and D). The affinity of *Af* L7Ae for a standard k‐turn has been measured in the pM range 42, so it is expected that we observe stoichiometric binding in these experiments, and consequently, we cannot measure a dissociation constant for this class of protein. However, collectively these data indicate that the 15.5k protein binds to and induces the folding of the box C/D and box C’/D’ k‐turns.

By contrast, the corresponding RNA species with N^6^‐methyladenine at the 1n position exhibited very different behavior. Methylated versions of both U13 and U62A RNA failed to form discrete retarded complexes observed by gel electrophoresis (Fig 4A and C), rather forming smears up the gels at high 15.5k concentrations, indicative of non‐specific binding. The fluorescence data (Fig 4B and D) show evidence of structural transitions at higher 15.5k concentration, with a significantly lower endpoint (final *E*_FRET_ = 0.3) compared to the unmethylated RNA. Moreover, the shape of the transition indicates rather greater cooperativity. These results suggest non‐specific binding of multiple protein molecules that fails to fold the methylated RNA into a normal k‐turn conformation.

We have explored the binding of 15.5k protein to SNORD62A (U62A) snoRNA with and without N6 methylation of A1n using isothermal titration calorimetry (Appendix Fig S1). Binding results in the evolution of heat in an exothermic reaction for both species, and the fitted thermodynamic data are tabulated in Appendix Table S2. The titration of the unmethylated RNA is consistent with stoichiometric binding as anticipated, with close to 1:1 15.5k:RNA ratio in an enthalpically driven binding reaction. However, the character of the binding to the N^6^A‐methylated RNA is different, having lower values of ∆H and T∆S and weaker apparent binding affinity, with a consequently different 15.5k protein–RNA ratio. This clearly reflects a significantly different protein binding mode between unmethylated and N^6^A‐methylated RNA, consistent with the results of the electrophoretic and FRET analysis, and confirming non‐specific binding when the snoRNA is methylated.

Evidently, N^6^‐methylation of A1n disrupts the proper binding and folding of box C/D and C’/D’ k‐turns by the human 15.5k protein.

### A structural basis for the effect of m^6^A on k‐turn folding

In order to understand the structural basis for the effect of N^6^‐methyladenine on k‐turn conformation, we have used X‐ray crystallography to investigate the structural effect of this modification on different base pairs involving adenine. Each species was formed by the hybridization of a self‐complementary strand that contains a 5‐bromocytosine nucleotide used to calculate phases for the diffraction in most cases. Crystallographic statistics are presented in Materials and Methods.

**Figure 5:**
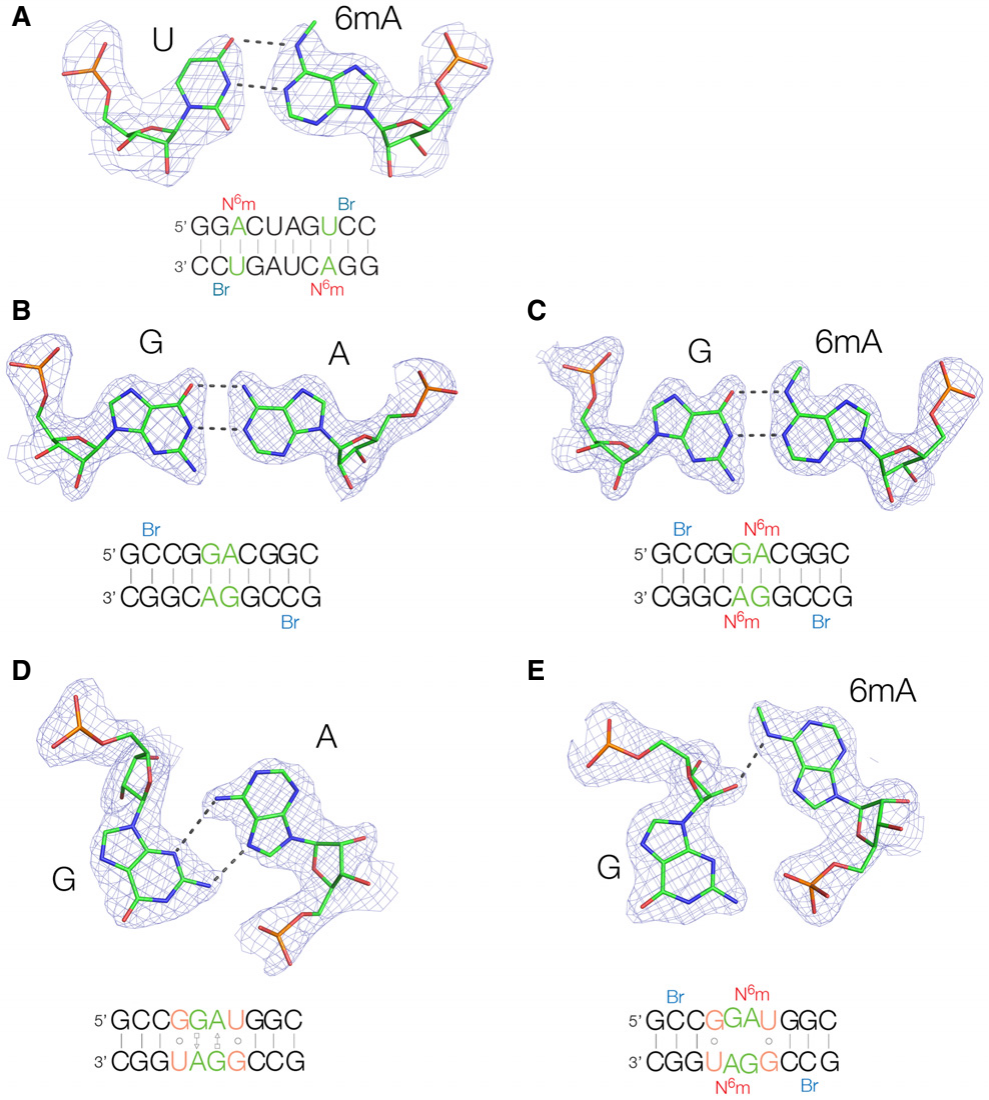
Crystal structures of duplex RNA containing base pairs involving adenine or N^6^‐methyladenine In each case, the relevant base pair is shown with its 2F_O_‐F_C_ map contoured at 1.2σ. The sequences of each self‐complementary duplex are shown, with the relevant base pair highlighted in green, and N^6^‐methyl modification in red. 5‐Bromocytosine nucleotides are shown blue; each structure was solved by SAD using the anomalous scatter from the two bromine atoms, except for 5LR3 that was determined by soaking with CuCl_2_. 
AA duplex containing a N^6^‐methyladenine‐uracil base pair (PDB 5LR5). Crystals of space group P6_5_ were obtained that diffracted to 2.27 Å. A standard *cis* Watson–Crick base pair is formed in this duplex, despite the presence of the N^6^‐methyl group.B, CAccommodation of N^6^‐methyladenine in a *cis* Watson–Crick G·A base pair. Two duplex species were constructed containing adjacent G·A base pairs flanked by G‐C base pairs, with (B, PDB 5LQO) and without (C, PDB 5LQT) N^6^‐methyladenine at 1.87 and 1.50‐Å resolution, respectively. In both structures, the G·A base pairs are *cis* Watson–Crick pairs connected by two hydrogen bonds. Thus, N^6^‐methylation of adenine does not lead to disruption of these Watson–Crick G·A pairs.D, EDisruption of a *trans* sugar‐Hoogsteen G·A base pair by N^6^‐methyladenine. Crystal structures of RNA duplexes in which guanine opposes adenine or N^6^‐methyladenine with flanking G·U base pairs. In the absence of the N^6^‐methyl group (D, PDB 5LR3 at 1.65‐Å resolution), a *trans* Hoogsteen‐sugar G·A base pair is formed. In marked contrast, the N^6^‐methyladenine does not form a base pair with the guanine (E, PDB 5LR4 at 1.72‐Å resolution), and there is no hydrogen bonding between the two nucleobases. Thus, N^6^‐methylation of adenine prevents the formation of the *trans* Hoogsteen‐sugar G·A base pair.

The first species studied contained N^6^‐methyladenine paired with uridine (PDB 5LR5). This sequence contains GGACU, which is methylated with the highest frequency in cellular RNA 9. The RNA duplex adopts standard A‐form helical geometry and the N^6^mA‐U base pairs are normal Watson–Crick pairs (Fig 5A). Formation of the *cis* Watson–Crick base pair requires that the N^6^‐methyl adopts an *anti* geometry to allow conventional basepairing with H‐bonding from AN6 to UO4. These results are in good agreement with recent NMR studies in solution of an RNA duplex from Kool *et al* 43.

We synthesized a second self‐complementary RNA strand that would hybridize to form central consecutive G·A pairs flanked by G‐C base pairs, with and without a methyl group on the adenine N6. The two species adopted closely similar structures (Fig 5B and C, Appendix Fig S2), with the G·A and G·6mA forming *cis* Watson–Crick base pairs in both cases that are well superimposed, irrespective of the presence of the methyl group on AN6. Like the A‐U base pair, the formation of the G·6mA base pair requires the *anti* isomer of the N^6^‐methyladenine. We conclude that N^6^‐methylation of adenine need not lead to disruption of the Watson–Crick G·A pairs.

**Figure 6:**
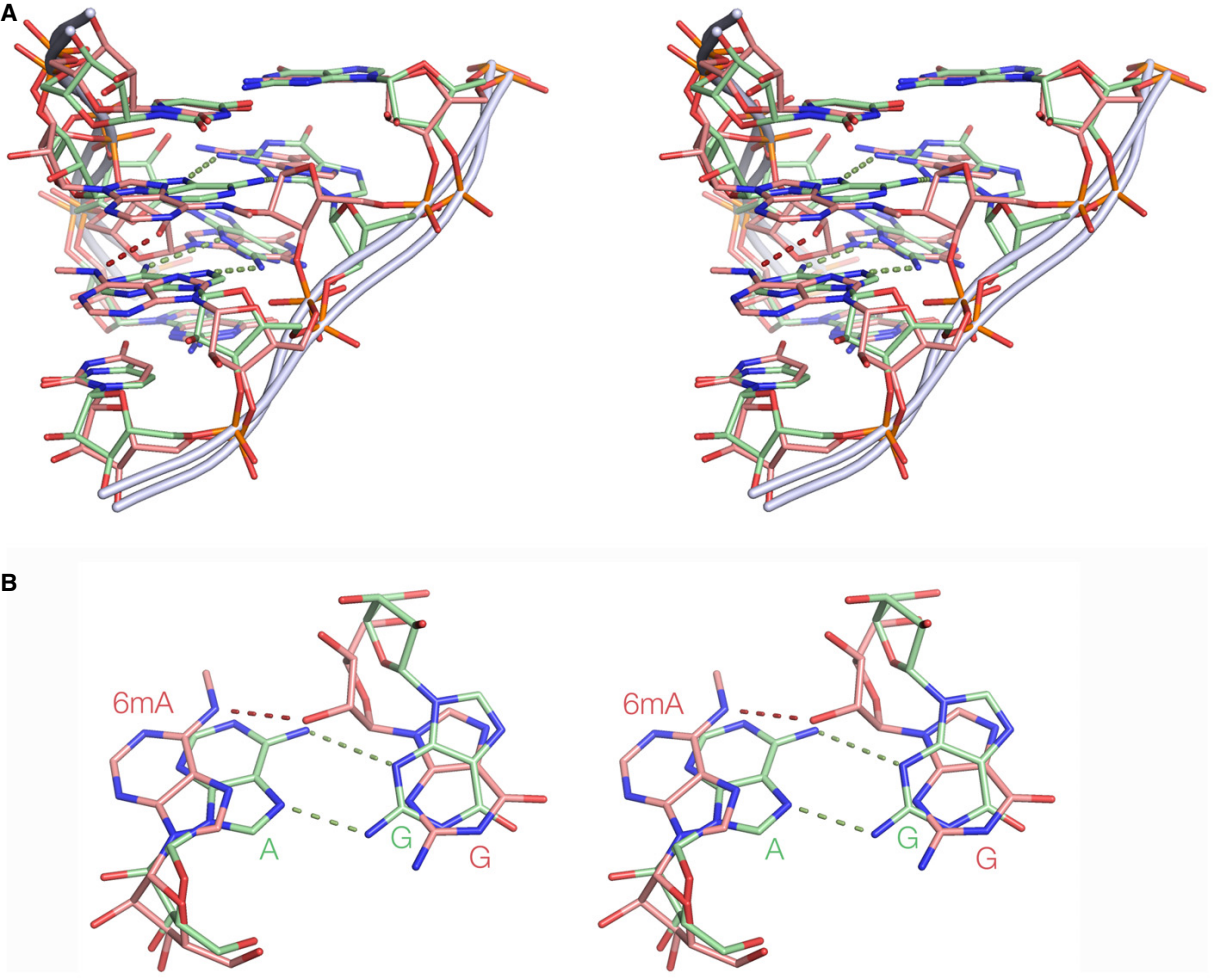
Superposition of RNA helices with potential *trans* sugar‐Hoogsteen G·A pairs (PDB 5LR3 and 5LR4), with and without N^6^‐methylation of adenine The unmethylated structure is shown (green) and the methylated structure (pink). Images are shown as parallel‐eye stereoscopic pairs. 
Stereo image of superimposed structures, showing the central G·A pairs and flanking G‐U base pairs.Superimposed view of one of the G·A pairs. Note that the G·N^6^mA base pair is disrupted, such that the N^6^‐methyladenine is significantly translated away from the opposing guanine base. The RMSD for the four G, A nucleotides is 0.962 Å.

We studied another duplex of sequence identical to that of the previous one, except that the G‐C base pairs flanking the central G·A pairs were replaced by G·U pairs (Fig 5D and E). Two forms were synthesized, with and without N^6^‐methyladenine replacing the central adenine bases that oppose the guanine nucleotides. Both were crystallized, and structures solved for the unmethylated RNA (PDB 5LR3) at 1.65‐Å resolution and the methylated RNA (PDB 5LR4) at 1.72 Å (Appendix Fig S2). The unmodified RNA formed a fully basepaired helix, but in contrast to the previous sequence, the G·A pairs formed *trans* sugar‐Hoogsteen base pairs with two H‐bonds between GN2:AN7 (2.8 Å) and AN6:GN3 (3.4 Å; Fig 5D). This is the same as the two *trans* sugar‐Hoogsteen G·A base pairs found in the core of the k‐turn. The flanking G·U pairs form *trans* base pairs connected by a single H‐bond GN2:UO4 (2.8 Å). In marked contrast, in the N^6^‐methyladenine‐containing duplex the guanine and N^6^‐methyladenine do not form base pairs (Fig 5E). Superposition (Fig 6) shows that it is primarily the N^6^‐methyladenine that has become displaced, with a large in‐plane translation so that the previously H‐bonded distances are now > 6 Å. This avoids a steric clash between the additional methyl groups and the ribose of the opposing guanine (AN6 is just 3 Å from the ribose in the *trans* sugar‐Hoogsteen G·A base pair). In its new position, AN6 donates a H‐bond to the O2′ of the opposing G (2.8 Å) and the methyl group is free from steric clash.

Thus, the effect of N^6^‐methylation of adenine in this context is the complete disruption of the *trans* sugar‐Hoogsteen base pair with guanine and is therefore incompatible with the k‐turn conformation. This can be seen if a methyl group is added to the N6 position of A1n in a standard k‐turn, whereupon the methyl can be seen to clash with the ribose of G1b (Appendix Fig S3). This provides a structural explanation for the observed failure of the k‐turn methylated at the A1n position to bind the 15.5‐kDa protein and adopt the k‐turn conformation.

## Discussion

N^6^‐methyladenine is the most common covalent modification of mRNA in eukaryotes. In principle, there are two ways of expressing N^6^‐methyladenine modification. One is recognition by a specific binding protein, frequently called a “reader” in the language of epigenetics. For example, He and colleagues have identified many Gm^6^AC sites in RNA that bind the YTHDF2 protein 9, 11. Alternatively, N^6^‐methylation of adenine could alter the local conformation of RNA. We have shown that the Watson–Crick base pairs A‐U and A‐G can form normally despite methylation of adenine at N6, although the A‐U base pair has been shown to be destabilized to some degree 43. In marked contrast, we have found that the *trans* Hoogsteen‐sugar A·G pair is completely disrupted by inclusion of N^6^‐methyladenine. This base pair is frequently found in RNA structures that are not regular duplex, and form the core of the k‐turn structure where the conserved adenine bases make key cross‐strand hydrogen bonds. We have shown here that this provides the basis for a probable regulatory mechanism for box C/D snoRNP assembly. The 15.5k protein fails to bind to the k‐turn when the A1n is methylated at the exocyclic N6. All the specific interactions between this class of protein and k‐turns are made with the conserved guanine nucleotides at the 1b and 2n positions that lie in the major groove on the outside of the kinked RNA, whereas the A1n is on the inside of the structure making no contact with the protein 35. This class of protein therefore makes indirect recognition of the methylation, resulting from the overall RNA structure rather than specific contacts. Recently, it has been shown that RNA structure can be affected *in vitro* by N^1^‐methylation of A or G 44, but we have not found evidence for such modification occurring naturally in box C/D snoRNA sequences.

The great majority of known k‐turn‐forming sequences have a C‐G base pair in the ‐1b, ‐1n position, so creating a GAG sequence on the unbulged strand that is not a target for N^6^‐methylation at A1n. This is true for almost all the known ribosomal and riboswitch k‐turn sequences. However, we have shown here that in a subset of human box C/D and C’/D’ snoRNA the ‐1b,‐1n base pair is reversed so placing a C at the ‐1n position, and thereby creating a GAC sequence that potentially targets the A1n for N^6^‐methylation. Approximately half of these have been shown (generally in multiple experiments) to be methylated *in vivo*. Importantly, the ‐1n = C is strongly conserved for those that are methylated, whereas this is not true for those that are not methylated.

We have shown here that N^6^‐methylation of A1n in human box C/D and C’/D’ k‐turns known to undergo methylation *in vivo* prevent the binding of the 15.5k protein and the concomitant folding of the k‐turn into the kinked conformation. This is the first stage of the assembly of the box C/D snoRNP, without which formation of an active methylation complex cannot proceed further 30. Although assembly factors are also involved 32, 45, 46, 47, the initial binding of 15.5k protein is fundamental, and blocking the required RNA structure is likely to be fatal to the assembly regardless. Thus, the assembly of these box C/D snoRNPs could plausibly be regulated by N^6^mA methylation of the guide RNA species. It has been shown that binding 15.5k protein stabilizes box C/D snoRNA 48, and if complex formation fails to occur, the RNA is unstable to degradation 49. In the absence of any of the core, snoRNP proteins box C/D snoRNA failed to accumulate in the nucleolar body of the nucleolus 50.

Is this restricted solely to box C/D snoRNA? 15.5k additionally binds the U4/U6.U5 tri‐snRNP in spliceosome assembly 51, where it also interacts with a k‐turn in the RNA 52 as the first stage of assembly 53. Given the similarities to box C/D snoRNP assembly, could this be subject to the same kind of regulation? In fact the U4 k‐turn sequence is standard with ‐1n = G, and this is strongly conserved from humans down to yeast. So this cannot be subject to regulation by methylation in the same way as the box C/D snoRNP species.

**Figure 7:**
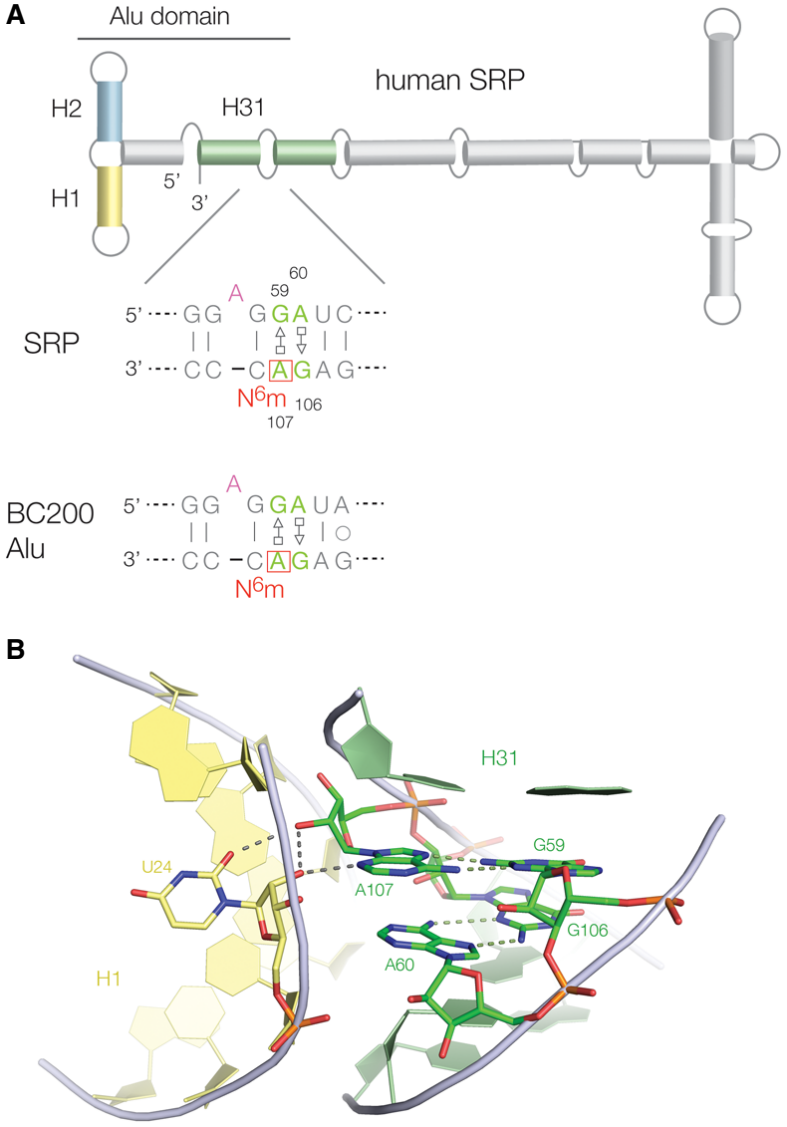
N^6^‐methylation of an adenine in human SRP, at a *trans* Hoogsteen‐sugar A·G base pair that mediates a tertiary contact A schematic showing the secondary structure of human SRP. Below is shown methylated sequence, and its equivalent in the Alu element BC200. Both have been shown to be N^6^‐methylated at the adenine boxed in red.The tertiary contact between helices 31 (green, containing the A·G pairs) and helix 1 (yellow) in the Alu domain of SRP, PDB 5AOX 58. The A·G pairs both form *trans* Hoogsteen‐sugar base pairs with the adenine bases directed into the minor groove of the H1 helix. A107 makes hydrogen bonding contacts between the two helices.

However, we have found data that are indicative of potential regulation by N^6^mA methylation at G·A base pairs in other systems. In fact one of the box C/D k‐turns we identified as methylated (SNORD71, HBII 239; Fig 3) has been shown to be a precursor of the human microRNA mir768 54 so connecting this with regulatory RNA species. Searching more widely we noted that the human signal recognition particle (SRP) RNA Alu domain contains a sequence that had been considered as a putative k‐turn, with the normal consecutive G·A and A·G base pairs (Fig 7). Moreover, like box C/D snoRNP, the SRP is assembled in the nucleolus 55, 56. This has a HS_2_HS_1_ structure 57, but the known *Haloarcula marismortui* k‐turn Kt‐58 has the same secondary structure and a closely similar sequence. The nucleotide immediately 3′ of the GA sequence of SRP on the unbulged strand (C108 on the lower strand as drawn in Fig 7) is strongly conserved as cytosine, so creating a potential GAC methylation target. Examination of a recent crystal structure of SRP 58 (Appendix Fig S4B) shows that while this sequence did not adopt k‐turn conformation, consecutive *trans* sugar‐Hoogsteen G·A and A·G base pairs were formed that directed the adenine nucleobases into the minor groove of the juxtaposed helix 1 to make an A‐minor interaction to stabilize the tertiary structure. A107 N3 accepts an H‐bond from the O2′ of U24, and its O2′ donates an H‐bond to O2 of U24 (Fig 7B). This is closely analogous to the A‐minor interactions found in the core of a standard k‐turn structure, but in this case, they are making a longer‐range tertiary contact. We examined the RMBase database 39 to see whether the central adenine (A107) is methylated *in vivo*. This revealed that this adenine has indeed been demonstrated to N^6^‐methylated in 14 independent experiments (Appendix Fig S4C). A107 would correspond to A1n were this forming a k‐turn and is the nucleotide that makes the key cross‐helix H‐bonds that fixes the tertiary structure (Fig 7B). From our crystallography, we know that N^6^‐methylation is incompatible with the required *trans* sugar‐Hoogsteen G·A base pair and that the adenine must translocate substantially. It is therefore most likely that methylation would destabilize the folded structure of the SRP.

Generalizing a little further, the left‐hand side of SRP (as depicted in Fig 7A) is closely related to the Alu retrotransposon, which is an extremely widespread human mobile genetic element. We examined one Alu element BC200 using RMBase and discovered that the equivalent adenine is indeed N^6^‐methylated *in vivo* (Appendix Fig S4). We further examined the first 30 human Alu elements (from a total of 1,862 entries) in the RMBase database and found that 11 were methylated at the corresponding adenine (i.e. the position corresponding to A1n in the k‐turn) (Appendix Fig S4C). Evidently, this adenine is subject to N^6^‐methylation at a high frequency in Alu elements in human cells.

In summary, crystallographic investigation shows that the major structural effect of N^6^‐methylation of adenine is likely to be manifest through the *trans* sugar‐Hoogsteen G·A sheared base pair rather than Watson–Crick base pairs. These are widely used in RNA structures, including rather prominently in k‐turns. We find that a subset of box C/D snoRNAs are methylated at the 1n position and that this blocks both 15.5k binding and the formation of the kinked conformation *in vivo*. But these effects are not restricted to k‐turns, and can apply to any structure that uses *trans* sugar‐Hoogsteen G·A base pairs, and we have found equivalent N^6^‐methylation of adenine in human SRP and in related Alu elements. It is likely that control of RNA conformation by N^6^‐methylation of adenine in G·A base pairs is quite general.

## Materials and Methods

### Bioinformatic analysis

#### Human box C/D k‐turn WebLogo

A full alignment of 262 of human box C/D sequences taken from snoRNABase 37 (https://www-snorna.biotoul.fr//) was made using Jalview (Jalview 2.9) 59. The k‐turn region was aligned manually based on the known pattern of conserved nucleotides. A WebLogo plot 60 showing the occurrence of box C/D sequences in human snoRNA was made using the website http://weblogo.threeplusone.com.

#### Identification of box C/D RNAs with a 2n = G, 1n = A, and ‐1n = C sequence

Human box C/D sequences extracted from snoRNABase 37 and snOPY 38 databases (http://snoopy.med.miyazaki-u.ac.jp) were checked with the Microsoft Word *search* function and visual alignment to identify box C/D RNAs with 2n = G, 1n = A, and ‐1n = C sequence.

#### ‐1nC conservation analysis

Box C/D sequences extracted from snoRNABase 37 and snOPY 38 databases were further aligned and manually inspected by Genedoc 61 and Jalview 59. Jalview was used to calculate the number and percentage of ‐1n = C. The quality of the alignment was also checked by comparison with a recent alignment of box C/D sequences deposited on Rfam 62, 63.

#### Identification of sites of N^6^‐adenine methylation using RMBase

Candidate box C/D snoRNAs were used to search RMBase 39 http://mirlab.sysu.edu.cn/rmbase/index.php in order to discover their A1n methylation status.

### RNA synthesis

RNA oligonucleotides were synthesized using *t*‐BDMS phosphoramidite chemistry 64 as described in Wilson *et al* 65, implemented on an Applied Biosystems 394DNA/RNA synthesizer. RNA was synthesized using ribonucleotide phosphoramidites with 2′O‐*tert*‐butyldimethyl‐silyl (*t*‐BDMS) protection 66, 67 (Link Technologies). *t*‐BDMS‐protected N^6^‐methyl‐A‐CE phosphoramidite was obtained from Glen Research. Fluorescein (Link Technologies) and Cy3 (GE Healthcare) were attached to the 5′ termini of the oligonucleotides as phosphoramidites in the final cycle of synthesis as required. Unmodified RNA was deprotected in a 25% ethanol/ammonia solution at room temperature for 3 h and evaporated to dryness. Oligonucleotides containing N^6^‐methyladenine were further deprotected for 2 h at 65°C. Oligonucleotides containing 5‐bromocytidine (ChemGenes) were deprotected for 36 h at 20°C. All oligoribonucleotides were redissolved in 115 μl of anhydrous DMSO, 60 μl triethylamine (Aldrich), and 75 μl triethylamine trihydrofluoride (Aldrich) to remove *t*‐BDMS groups, and agitated at 65°C in the dark for 2.5 h. After cooling on ice for 10 min, 250 μl RNA quenching buffer (Glen Research) was added to stop the reaction, and the oligonucleotides were desalted using NAP‐10 columns (GE Healthcare).

RNA for crystallization was purified by gel electrophoresis in polyacrylamide under denaturing conditions in the presence of 7 M urea. The full‐length RNA product was visualized by UV shadowing. The band was excised and electroeluted using an Elutrap Electroelution System (GE Healthcare) into 45 mM Tris‐borate (pH 8.5), 5 mM EDTA buffer for 8 h at 200 V at 4°C. The RNA was precipitated with ethanol, washed once with 70% ethanol, and suspended in water.

Fluorophore‐labeled oligoribonucleotides were purified by gel electrophoresis under denaturing conditions (as described below) and subjected to further purification by reversed‐phase HPLC on a C18 column (ACE 10‐300, Advanced Chromatography Technologies), using an acetonitrile gradient with an aqueous phase of 100 mM triethylammonium acetate (pH 7.0). Duplex species used for FRET experiments were prepared by mixing equimolar quantities of the appropriate oligoribonucleotides and annealing them in 90 mM Tris‐borate (pH 8.5), 10 mM EDTA, 25 mM NaCl, by slow cooling from 90°C to 4°C. They were purified by electrophoresis in 12% polyacrylamide under nondenaturing conditions and recovered by electroelution, followed by ethanol precipitation.

### Preparation of proteins

#### L7Ae protein

*Archeoglobus fulgidus* L7Ae protein was expressed and purified as previously published 35.

#### 15.5‐kDa protein

Human 15.5k protein was expressed as an N‐terminal GST fusion in *Escherichia coli* BL21 (DE3) pLysS induced with 0.2 mM isopropyl β‐D‐1‐thiogalactopyranoside at 22°C. Harvested cells were resuspended in PBS buffer and lysed by sonication. Insoluble protein was removed by ultracentrifugation at 45,000 *g* for 60 min at 4°C. The solution was then applied to Glutathione Sepharose 4B resin (GE Healthcare) equilibrated in buffer PBS. The GST tag was cleaved by incubation with 40 U/mg thrombin in 20 mM Tris–HCl (pH 8.0), 50 mM NaCl for 8 h at 25°C. In order to remove contaminating RNA the protein was applied to a heparin column (GE Healthcare) in 20 mM Tris–HCl (pH 8.0) with a 50–2,000 mM NaCl gradient; 15.5k protein eluted at 500 mM NaCl. The purified protein was concentrated to 10 mg/ml.

### FRET analysis of k‐turn folding

Fluorescence resonance energy transfer efficiency was measured from duplex RNA terminally 5′‐labeled with fluorescein and Cy3. RNA duplexes contained a central box C/D sequence or N^6^‐A1n‐substituted variant (from position −2 to 4). The sequences used were (written 5′–3′):

U13top: F –CCAGUCAGUGAGCGUGAUGCAUGUCAGG
U62Atop: F –CCAGUCAGUGGGAGAUGAAGCAUGUCAGG
U48top: F –CCAGUCAGUGGUGAUGAUGCAUGUCAGG
lower strand: Cy3‐CCUGACAUGCUGACCCACUGACUGG

Note that the lower strand is the same in each case.

Absorption spectra were measured in 90 mM Tris‐borate (pH 8.3) in a 5‐mm path‐length cuvette in 2 μl volumes using a NanoDrop 2000c spectrophotometer (Thermo Scientific). Spectra were deconvoluted using a corresponding RNA species labeled only with Cy3, and fluorophore absorption ratios calculated using a MATLAB program. Fluorescence spectra were recorded in 90 mM Tris‐borate (pH 8.3) at 4°C using an SLM‐Aminco 8100 fluorimeter. Spectra were corrected for lamp fluctuations and instrumental variations, and polarization artifacts were avoided by setting excitation and emission polarizers crossed at 54.7°. Values of FRET efficiency (*E*_FRET_) were measured using the acceptor normalization method 68 implemented in MATLAB.

*E*_FRET_ as a function of 15.5k protein concentration was fitted to:

(1) $$E_{FRET}=E_{0}+\Delta E_{FRET}\cdot \frac{\left(1+K_{A}P_{T}+K_{A}R_{T}\right)-\sqrt{{\left(1+K_{A}P_{T}+K_{A}R_{T}\right)}^{2}-4R_{T}K_{A}^{2}P_{T}}}{2R_{T}K_{A}}$$

where *E*_*0*_ is the initial FRET efficiency in the absence of added protein, *∆E*_FRET_ is the full range of the change in FRET efficiency, *K*_*A*_ is the apparent association constant, and *P*_*T*_ and *R*_*T*_ are the total concentration of 15.5k and RNA, respectively.

### Electrophoretic analysis of protein binding to snoRNA k‐turns

Binding of recombinant 15.5k and AfL7Ae protein to the U13 and U62A k‐turn motif RNA was analyzed by gel electrophoresis. 200 nM Cy3‐labeled RNA (the same species as used in the FRET experiments, see above) was incubated in 45 mM Tris‐borate (pH 8.3) with various concentrations of proteins (15.5k or AfL7Ae) in a final volume of 10 μl at 7°C for 60 min. An equal volume of loading buffer containing 10% glycerol and 45 mM Tris‐borate (pH 8.3) was added to the reactions, which were then electrophoresed in 10% native polyacrylamide gels in 45 mM Tris‐borate (pH 8.3) at 20 V/cm at 7°C for 1 h. Fluorescent RNA was visualized using a Typhoon 9500 fluorimager (GE Healthcare).

### Isothermal titration calorimetry

Titrations were performed at 298 K using an ITC‐200 microcalorimeter (GE). RNA solutions were prepared by diluting concentrated stocks into the binding buffer containing 40 mM HEPES (pH 7.5), 100 mM KCl, 10 mM MgCl_2_ to a final concentration of 20 μM. 15.5k was prepared in the same binding buffer with a concentration of 200 μM. Solutions were degassed for 2–5 min before loading. The sample cell was filled with 200 μl of RNA. 15.5k protein was injected into the RNA solution, using 0.4 μl for the first injection and 2 μl for the subsequent 19 injections using a computer‐controlled 40‐μl microsyringe with an injection interval of 120 s. Titration of protein into the binding buffer or titration of the binding buffer into the RNA solution produced negligible heat evolution. Integrated heat data were analyzed using a one‐set‐of‐sites model in MicroCal Origin following the manufacturer's instructions. The first data point was excluded in the analysis. The binding parameters Δ*H* (cal mol^−1^), *K* (M^−1^) and *n* (moles of 15.5k bound per RNA) were fitted. The binding free energy Δ*G* and reaction entropy Δ*S* were calculated using the relationships Δ*G* = −*RT* ln *K* (*R* = 1.9872 cal mol^−1^ K^−1^, *T* = 298 K) and Δ*G* = Δ*H* − *TΔS*. The dissociation constant *K*_d_ was calculated as 1/*K*.

### Crystallization, structure determination, and refinement

The sequences are shown in Fig 4. All RNA used in crystallization in this study was formed by hybridization of self‐complementary strands. A solution of 1 mM RNA in 5 mM Tris–HCl (pH 8.0), 100 mM NaCl was heated to 95°C for 1 min. The solution was slowly cooled to 20°C, and MgCl_2_ was added to a final concentration of 10 mM. The complete conditions used for crystallization of each species are given in Appendix Table S3.

All data were collected on beamline I02 of Diamond Light Source (Harwell, UK), and processed by XIA2 version 0.4.0.0 69. The resolution cutoff for the data was determined by examining by CC_1/2_ and density map as described previously 70.

All the structures’ initial phases were acquired from the SAD data by locating the bromine atoms with Autosol in the PHENIX suite, except for 5LR3 that was determined by soaking with CuCl_2_. Models were adjusted manually using Coot 71 and subjected to several rounds of adjustment and optimization using Coot, phenix.refine and PDB_REDO 72. Model geometry and the fit to electron‐density maps were monitored with MOLPROBITY 73 and the validation tools in Coot. Data collection and refinement statistics are presented in Appendix Table S4.

### Data availability and accession numbers

Coordinates of the crystal structures described in this work are deposited with the PDB with accession numbers 5LR5, 5LQO, 5LQT, 5LR3, and 5LR4.

## Author contributions

LH performed bioinformatic analysis and crystallography, SA carried out RNA synthesis, JW performed bioinformatic analysis, and LH and DMJL planned experiments, analyzed data and wrote the paper.

## Conflict of interest

The authors declare that they have no conflict of interest.

## Supporting information



Appendix

Review Process File
