# Supplementary material for: Control of box C/D snoRNP assembly by N6‐methylation of adenine
Source: EMBO Rep. 2017 Jun 16;18(9):1631–45. doi: 10.15252/embr.201743967 (PMC5579392; doi:10.15252/embr.201743967)
Supplement: Supplementary file 1 — Appendix [file EMBR-18-1631-s001.pdf]

Control of box C/D snoRNP assembly by N^6-methylation of adenine

by L. Huang, S. Ashraf, J. Wang and D. M. J. Lilley

APPENDIX

2. Appendix Figure S1. Analysis of 15.5k protein binding to the 4 by isothermal titration calorimetry.

3. Appendix Figure S2. Crystal structures of duplex RNA containing A•G pairs without and with N^6 methylation of adenine

4. Appendix Figure S3. Potential steric clash by a methyl group added to A1n N6 in Kt-7.

5. Appendix Figure S4. N^6-methylation of an adenine in human SRP, at a *trans* Hoogsteen-sugar A•G basepair that mediates a tertiary contact.

6. Appendix Table S1. Human box C/D snoRNA sequences conforming to 2n = G, 1n = A, and -1n = C sequences,

7. Appendix Table S2. Thermodynamic data on the binding of 15.5k protein to U62A box C'/D' RNA obtained by isothermal titration calorimetry.

8. Appendix Table S3. Crystallization conditions used for RNA species with and without N^6-methyladenine

9. Appendix Table S4. Details of data collection and refinement statistics for the data as deposited in the PDB.

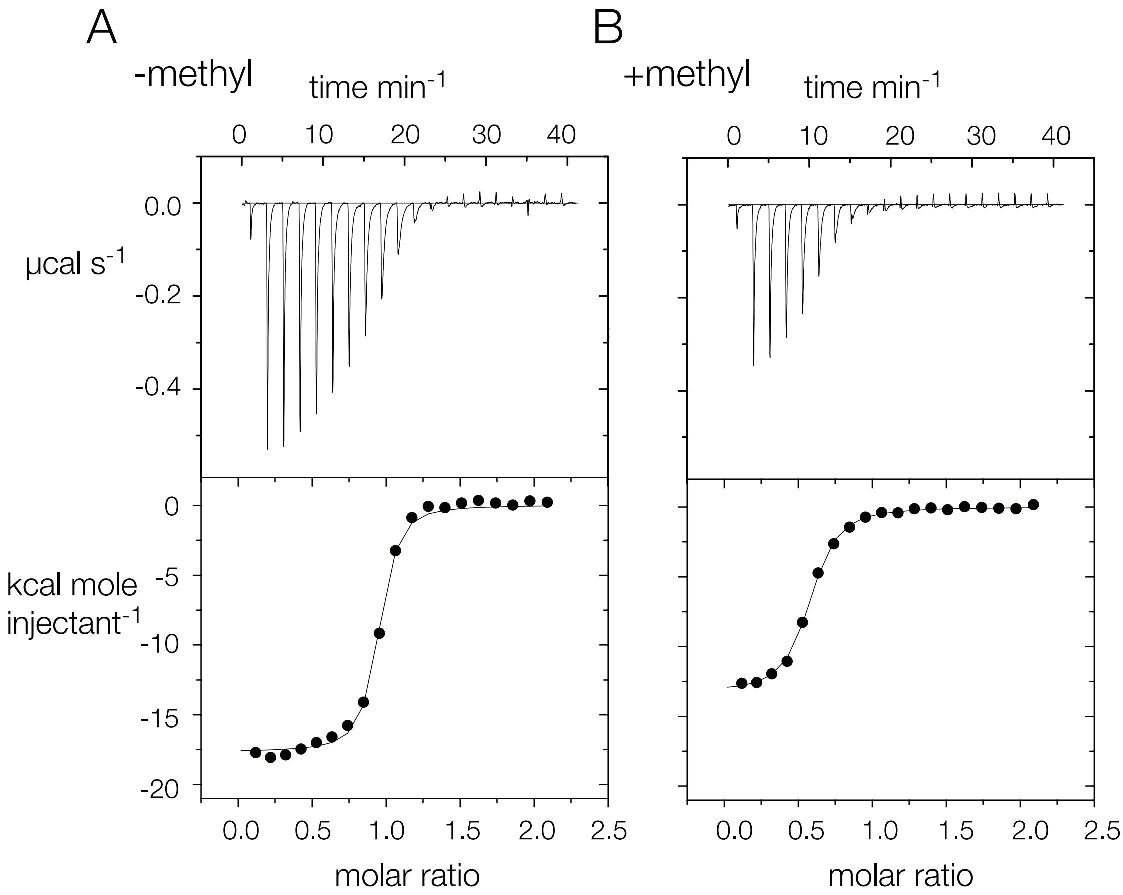

Appendix Figure S1. Analysis of 15.5k protein binding to the 4 by isothermal titration calorimetry. 15.5k protein at 200 μM was injected into 20 μl of a solution of U62A box C'/D' RNA with a concentration of 200 μM at 298 K. The initial injection was 0.4 µl followed by 2 µl for the next 19 injections with an injection interval of 120 s, in a buffer containing 40 mM HEPES (pH 7.5), 100 mM KCl, 10 mM $MgCl_2$. The analysis was performed for U62A box C'/D' RNA without (**A**) or with (**B**) N^6 methylation at the A1n position. The data were fitted and the thermodynamic parameters tabulated in Appendix Table S2.

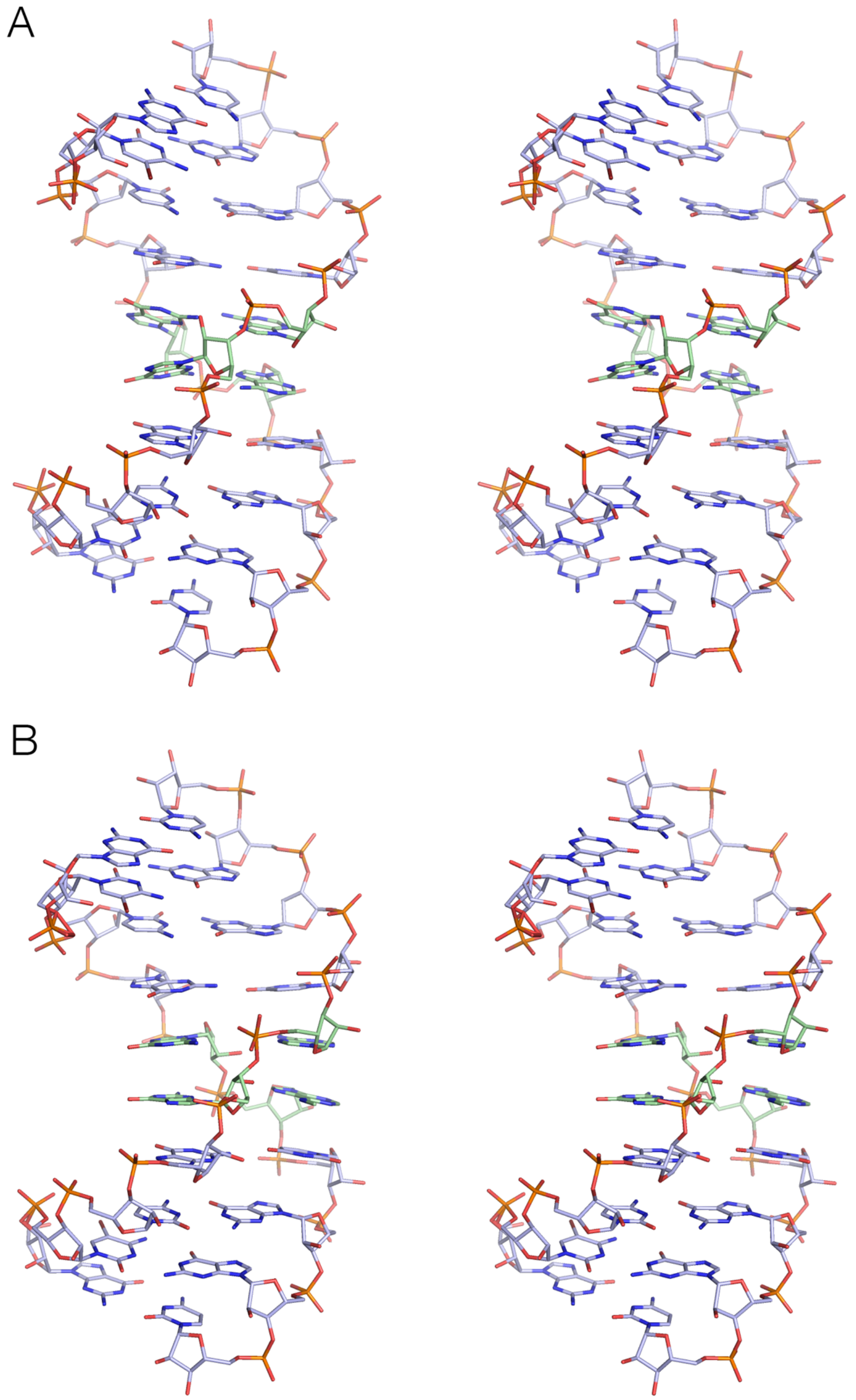

Appendix Figure S2. Crystal structures of duplex RNA containing A•G pairs without (**A**) and with (**B**) N^6 methylation of adenine (PDB 5LR3 and 5LR4). The complete 10 bp duplexes are shown as parallel-eye stereoscopic pairs. with A•G pairs drawn in green.

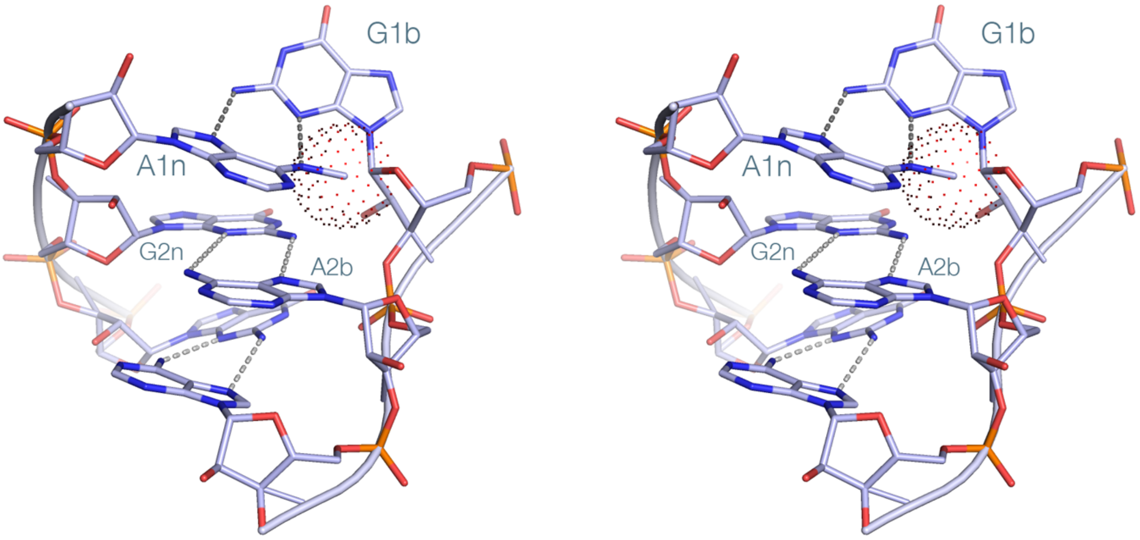

Appendix Figure S3. Potential steric clash by a methyl group added to A1n N6 in Kt-7 (PDB 4CS1), a standard k-turn structure. A parallel-eye stereoscopic view is shown, with the volume occupied by the methyl group indicated by the dotted region.

A

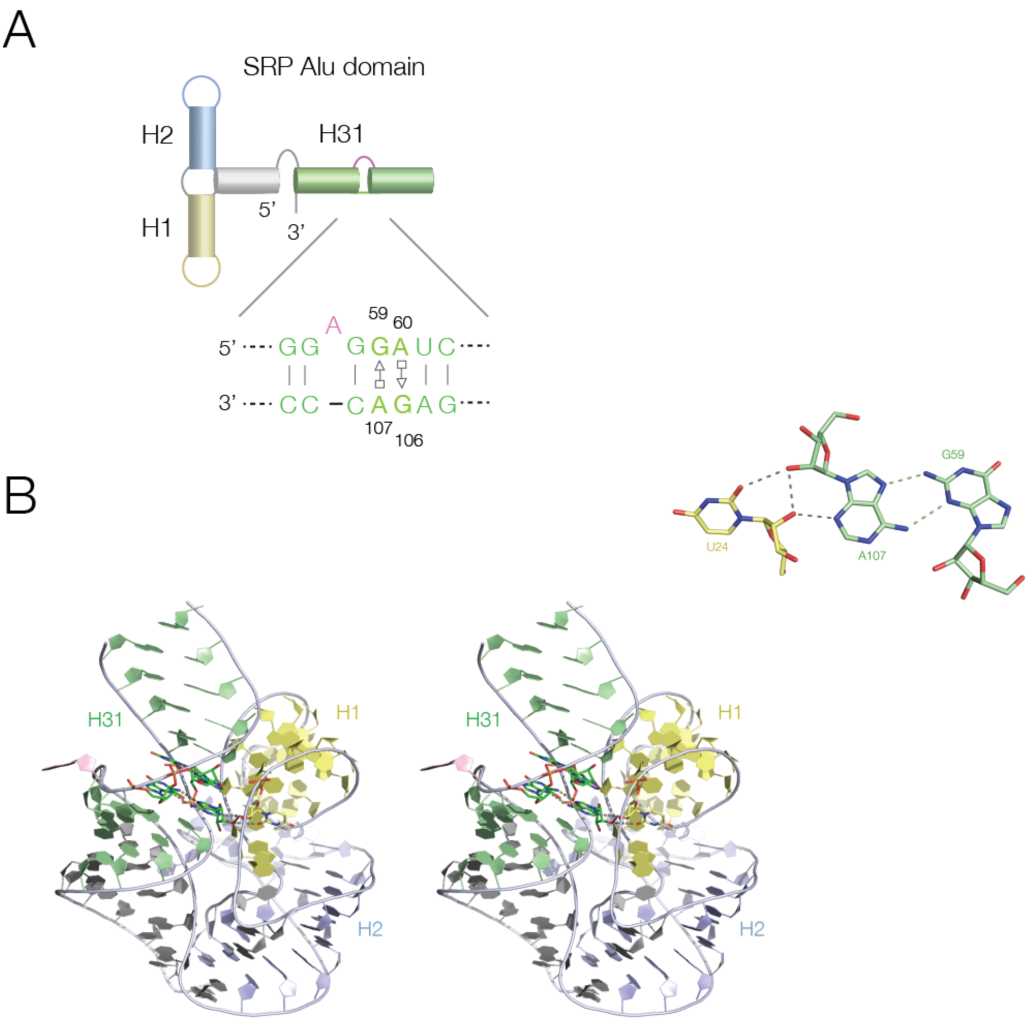

C

gene	mod ID	xsome	position	support no	sequence (N^6meA)
srpRNA	m6A_site_38024	chr14	50329278	14	AGCCTGAGCAACATAGCGAGACCCCGTCTCTTTTGCCCCCC
AluJo	m6A_site_12507	chr1	214372362	5	GCCTGAGCAACATAGCGAGACCCCGTCTCTAAAACAAAAA
AluSp	m6A_site_9026	chr1	154184358	4	GCCTGGGCAACAAAAGTGAAACTCTGTCTCAAAAAAAAAAA
FAM	m6A_site_31320	chr12	76858065	4	AGCCTGAGCAACATAGTGAGACCCTGTCTCTAAAAAGCAAC
AluJb	m6A_site_32596	chr12	112742257	4	AGCCTGGGCAACATAGCGAGACCCCGTCTCTGCAAAAAAAT
AluJr	m6A_site_119490	chr7	70342837	4	AGCCTGAGCAACATAGCGAGACCCCGTCTCTACAAAAACTA
AluSc	m6A_site_134766	chr9	139728960	4	TCCAGCCTGGACAGAGTGAGACTCCGTCTCCAAAAAAAAGA
AluJr	m6A_site_30705	chr12	58271974	3	AGCCTGAGCAACATAGCGAGACCCCGTCTCTACAAAAAAAA
AluSx3	m6A_site_30910	chr12	66517102	3	AGCCTGGCTAATACAGTGAAACCCCATCTCTACTAAAAATA
AluSz6	m6A_site_40909	chr14	104057184	3	AGCCTGGAAGACCAAACGAGACTCTGTATCAAAAAATAATA
AluSz	m6A_site_62849	chr19	2342640	3	AGCCTGGGTGACAGAGTGAGACTCCATCTCAAAAACAAAAT
AluSz	m6A_site_64656	chr19	12986232	3	AGCCTGGGTGACAGAATGAGACTCTGTGTCAAAAAAATAAA
BC200	m6A_site_139606	chrX	138802845	1	TGCCTGGGCAATATAGCGAGACCCCGTTCTCCAGAAAAAGG

Appendix Figure S4. N^6-methylation of an adenine in human SRP, at a *trans* Hoogsteen-sugar A•G basepair that mediates a tertiary contact.

A. Schematic of the secondary structure of the human SRP Alu domain, with the secondary structure of the section containing the G•A, A•G basepairs.

B. The structure of the SRP Alu domain, PDB 5AOX [1] shown as a parallel-eye stereoscopic pair. The tertiary interaction between helices 31 and 1 mediated by A107 is shown.

C. RMBase database [2] output showing N^6-methyladenine sites in the human SRP and 11 human Alu elements. The position of the N^6-methyladenine is shown in red.

snoRNA name	Rfam ID	range	box	me	target RNA	C-1n conservation	
						snOPY	snoRNAbase
SNORD101 (U101)	RF00186	Vertebrates	D	+	Unknown	26 of 26 (100%)	
SNORD13	RF01210	Vertebrates	D	+	Unknown		9 of 12 (75.00%)
SNORD2 (snR39B)	RF01299	Vertebrates	D'	+	28S rRNA; G1509		14 of 16 (87.50%)
SNORD29 (U29)	RF00070	Tetrapodes and Teleostes	D'	+	28S rRNA; A4493	20 of 28 (71.42%)	
SNORD41 (U41)	RF00588	Eutherians	D'	+	28S rRNA; U4276	20 of 21 (95.23%)	
SNORD44 (U44)	RF00287	Tetrapodes and Teleostes	D'	+	18S rRNA; A166	19 of 20 (95.00%)	
SNORD46 (U46,U40)	RF00218	Vertebrates	D	+	28S rRNA; A3739	20 of 22 (90.90%)	
SNORD48 (U48)	RF00282	Tetrapodes and Teleostes	D	+	28S rRNA; C2279	23 of 24 (95.83%)	
SNORD5 (mgh28S-2409)	RF01161	Amniotes	D	+	28S rRNA; C2409		9 of 13 (69.23%)
SNORD57(U 57)	RF00274	Amniotes	D'	+	18S rRNA; A99	25 of 26 (96.15%)	
SNORD62A (U62A)	RF00153	Amniotes	D'	+	18S rRNA; A590		
SNORD67 (HBII-166)	RF00573	Deuterostomes	D	+	U6 snRNA; C60	21 of 23 (91.30%)	14 of 15 (93.30%)
SNORD71 (HBII-239)	RF00576	Amniotes	D	+	5.8S rRNA; U14	8 of 8 (100%)	26 of 26 (100%)
SNORD89 (HBII-289)	RF00578	Deuterostomes	D	+	Unknown	22 of 23 (95.65%)	
SNORD105 B (U105B)	RF01173	Eutherians	D	-	18S rRNA	1 of 19 (5.26%)	8 of 22 (36.36%)
SNORD111 (HBII-82)	RF00611	Amniotes	D'	-	28S rRNA	19 of 50 (38.00%)	
SNORD118	RF00096	Eutherians	D	-	28S rRNA	1 of 3 (33.33%)	
SNORD18B (U18B)	RF00093	Deuterostomes	D	-	28S rRNA	14 of 109 (12.84%)	
SNORD38B (U38B)	RF00212	Vertebrates	D'	-	28S rRNA	28 of 69 (40.58%)	
SNORD43L1	RF00221	Deuterostomes	D	-	Unknown		
SNORD44 (U44)	RF00287	Tetrapodes and Teleostes	D	-	18S rRNA	5 of 20 (25.00%)	
SNORD49B (U49B)	RF00277	Vertebrates	D	-	28S rRNA		8 of 10 (80.00%)
SNORD4B (Z17B)	RF00266	Amniotes	D	-	18S rRNA	2 of 5 (40.00%)	
SNORD60L1	RF00271	Tetrapodes and Teleostes	D'	-	Unknown		

Appendix Table S1. Human box C/D snoRNA sequences conforming to 2n = G, 1n = A, and -1n = C sequences, showing the conservation of cytosine at -1n.

RNA	n	ΔH / kJ.mol^{-1}	ΔS / J.K^{-1}.mol^{-1}	ΔG / kJ.mol^{-1}	K_d / nM
no methyl	0.91 ± 0.01	-17.64 ± 0.17	-26.8	-9.65 ± 0.17	82.6 ± 6.8
N^{6}mA1n	0.55 ± 0.01	-13.29 ± 0.12	-14.8	-8.88 ± 0.12	314.4 ± 22.8

Appendix Table S2. Thermodynamic data on the binding of 15.5k protein to U62A box C'/D' RNA obtained by isothermal titration calorimetry (see Appendix Figure S1). Because of the affinity of 15.5k for the RNA, the calculated value of K_d is not reliable.

type	PDB ID	vapor diffusion method	mother liquor	cryoprotectant
cis Watson-Crick m^6A-U	5LR5	sitting-drop	0.2 M $(NH_4)_2SO_4$, 0.1 M Na acetate trihydrate (pH 4.6) and 25% w/v polyethylene glycol 4000	mother liquor containing 20% w/v polyethylene glycol 400
cis Watson-Crick G•A	5LQO	hanging-drop	0.025 M $MgSO_4$, 0.05 M Tris.HCl (pH 8.5) and 1.8 M $(NH_4)_2SO_4$	mother liquor containing 30% glycerol
cis Watson-Crick G•m^6A	5LQT	hanging-drop	0.02 M $CaCl_2$ dihydrate, 0.1 M Na acetate trihydrate (pH 4.6), 30% v/v 2-methyl-2,4-pentanediol	mother liquor
trans sugar-Hoogsteen G•A	5LR3	hanging-drop	0.015 M Mg acetate, 0.05 M Na cacodylate (pH 6.0), 1.7 M $(NH_4)_2SO_4$	mother liquor containing 30% glycerol
unpaired G, m^6A	5LR4	hanging-drop	0.1M NaCl, 0.1M bis-Tris (pH 6.5), 1.5 M $(NH_4)_2SO_4$	mother liquor containing 30% glycerol

Appendix Table S3. Crystallization conditions used for RNA species with and without N^6-methyladenine.

	cis Watson-Crick m6A-U	*cis* Watson-Crick G•A	*cis* Watson-Crick G•m6A	*trans* sugar-Hoogsteen G•A		unpaired G, m6A
	5LR5	5LQO	5LQT	5LR3		5LR4
**Data collection**						
Space group	$P6_5$	$P4_3\ 2_1\ 2$	$P4_3\ 2_1\ 2$	$P4_3\ 2_1\ 2$	$P4_3\ 2_1\ 2$	$P4_3\ 2_1\ 2$
Cell dimensions						
a, b, c (Å)	50.47, 50.47, 108.63	33.47, 33.47, 113.77	33.34, 33.34, 103.17	33.30, 33.30, 120.91	33.65, 33.65, 117.75	49.87, 49.87, 123.73
α, β, γ (°)	90, 90, 120	90.0, 90.0, 90.0	90.0, 90.0, 90.0	90.0, 90.0, 90.0	90.0, 90.0, 90.0	90.0, 90.0, 90.0
	SAD-Br	SAD-Br	SAD-Br		SAD-Cu	SAD-Br
	Peak	*Peak*	*Peak*	Native	*Peak*	*Peak*
Wavelength	0.9193	0.9196	0.9193	0.9796	1.3776	0.9196
Resolution (Å)	43.71- 2.27 (2.31 - 2.27)*	33.47- 1.87 (1.92 – 1.87)*	33.35- 1.50 (1.53 – 1.50)*	29.17 – 1.65 (1.69 – 1.65)*	29.44-2.48 (2.54 - 2.48)*	46.25 – 1.72 (1.76 – 1.72)*
R_{merge}	0.143 (2.72)	0.072 (2.08)	0.056 (2.08)	0.063 (1.97)	0.069 (0.669)	0.075 (2.23)
$I / \sigma I$	13.3 (1.4)	10.7 (1.3)	21.7 (1.2)	16.8 (1.0)	32.6 (4.8)	9.5 (1.0)
CC (1/2)	1.00 (0.55)	1.00 (0.45)	1.00 (0.50)	1.00 (0.60)		1.00 (0.90)
Completeness (%)	98.4 (97.6)	100 (100)	100 (100)	98.5 (95.2)	99.0 (99.4)	100 (100)
Redundancy	41.7 (38.7)	12.2 (13.0)	11.9 (12.4)	9.9 (10.2)	21.1 (22.9)	11.7 (12.2)
**Refinement**						
Resolution (Å)	43.71 – 2.40	32.11 – 1.87	32.58 – 1.50	29.17 – 1.65		46.25 – 1.80
	(2.64 – 2.40)	(2.14- 1.87)	(1.58 – 1.50)	(1.77 – 1.65)		(1.87 - 1.80)
No. reflections	5911 (589)	5654 (552)	10522 (529)	8701 (802)		26766 (3076)
R_{work} / R_{free}	0.261 / 0.286	0.245 / 0.255	0.219 / 0.228	0.247 / 0.266		0.269 / 0.288
No. atoms						
Macromolecules	856	432	436	432		872
Solvent	15	20	56	38		60
B-factors						
Macromolecules	68.6	45.80	38.82	44.04		48.30
Solvent	66.8	45.77	43.35	42.11		47.10
R.m.s. deviations						
Bond lengths (Å)	0.011	0.006	0.011	0.100		0.010
Bond angles (°)	0.61	0.93	0.89	0.34		1.18

*Values in parentheses are for highest-resolution shell.

Appendix Table S4. Details of data collection and refinement statistics for the data as deposited in the PDB.

References

1. Ahl V, Keller H, Schmidt S, Weichenrieder O (2015) Retrotransposition and crystal structure of an Alu RNP in the ribosome-stalling conformation. *Molec cell* **60**: 715-727
2. Sun WJ, Li JH, Liu S, Wu J, Zhou H, Qu LH, Yang JH (2016) RMBase: a resource for decoding the landscape of RNA modifications from high-throughput sequencing data. *Nucleic Acids res* **44**: D259-265
